# Risk assessment models for genetic risk predictors of lung cancer using two-stage replication for Asian and European populations

**DOI:** 10.18632/oncotarget.10403

**Published:** 2016-07-05

**Authors:** Yang Cheng, Tao Jiang, Meng Zhu, Zhihua Li, Jiahui Zhang, Yuzhuo Wang, Liguo Geng, Jia Liu, Wei Shen, Cheng Wang, Zhibin Hu, Guangfu Jin, Hongxia Ma, Hongbing Shen, Juncheng Dai

**Affiliations:** ^1^ Department of Epidemiology and Biostatistics, School of Public Health, Nanjing Medical University, Nanjing, 211166, China; ^2^ Jiangsu Key Lab of Cancer Biomarkers, Prevention and Treatment, Collaborative Innovation Center of Cancer Medicine, Nanjing Medical University, Nanjing, 211166, China

**Keywords:** lung cancer, polymorphism, genetic risk score, risk prediction, ethnic populations

## Abstract

In the past ten years, great successes have been accumulated by taking advantage of both candidate-gene studies and genome-wide association studies. However, limited studies were available to systematically evaluate the genetic effects for lung cancer risk with large-scale and different ethnic populations. We systematically reviewed relevant literatures and filtered out 241 important genetic variants identified in 124 articles. A two-stage case-control study within specific subgroups was performed to assess the effects [Training set: 2,331 cases vs. 3,077 controls (Chinese population); testing set: 1,937 cases vs. 1,984 controls (European population)]. Variable selection and model development were used LASSO penalized regression and genetic risk score (GRS) system. Further change in area under the receiver operator characteristic curves (AUC) made by the epidemiologic model with and without GRS was used to compare predictions. It kept 38 genetic variants in our study and the ratios of lung cancer risk for subjects in the upper quartile GRS was three times higher compared to that in the low quartile (odds ratio: 4.64, 95% CI: 3.87–5.56). In addition, we found that adding genetic predictors to smoking risk factor-only model improved lung cancer predictive value greatly: AUC, 0.610 versus 0.697 (*P* < 0.001). Similar performance was derived in European population and the combined two data sets. Our findings suggested that genetic predictors could improve the predictive ability of risk model for lung cancer and highlighted the application among different populations, indicating that the lung cancer risk assessment model will be a promising tool for high risk population screening and prediction.

## INTRODUCTION

Lung cancer is one of the most commonly diagnosed malignancies and the leading cause of cancer-related deaths in the world, with almost 1.6 million deaths per year (19.4% of total cancer mortality) [1]. As well as known that the major environmental cause is tobacco smoking accounting for over 80% of all lung cancer cases. However, only less than 20% of smokers developed lung cancer cases, suggesting that individual variation in genetic susceptibility may play an important role [2]. Over the past ten years, both candidate-gene studies and genome-wide association studies (GWAS) have successfully identified dozens of loci associated with lung cancer risk. Although researchers have tested whether genetic variants identified from previous papers increased the models’ predictive ability of such common disorders: cardiovascular disease [3], breast cancer [4, 5], prostate cancer [6–8] and diabetes [9], limited studies were available to systematically evaluate the genetic effects for lung cancer risk with large scale populations [10–12].

Despite significant advances in medical therapy, prognosis of lung cancer remains poor with a five-year survival rate of 16.6% [13], as most cases are diagnosed at advanced stage. Indeed, when lung cancer is detected before metastasis, the five-year survival rates should be 60–80% [14]. Therefore, early detection and diagnosis for lung cancer was the focus of our future research. In this respect, screening high risk population of lung cancer is an important element.

As a result, we systematically reviewed all the relevant literatures and screened out the genetic variants associated with lung cancer risk. Then we performed a two-stage case-control design with nearly ten thousand samples to assess the effects of selected genetic predictors This study showed that genetic predictors could improve the predictive ability of risk model for lung cancer among different populations, facilitating the clinical and public health.

## RESULTS

### General description of subjects

NJMU GWAS contains 2 331 lung cancer cases and 3 077 healthy controls, which was used as the training set to construct the model, while EAGLE study containing 1 937 cases and 1 984 controls was used to validate the model. Compared to controls with 52.66% smoking rate, cases had a significantly higher rate of smoking with 76.85% among the two data sets (Supplementary Table S1).

### General information of genetic risk score

Forty of 241 lung cancer-associated SNPs were statistically significantly associated with lung cancer risk in this study at *P* less than 0.05 through univariate analysis (data not shown). Further, LASSO penalized regression based on univariate analysis selected 38 SNPs in the training set as shown in Table 1. To assess the cumulative risk values for the genetic predictors, we calculated a “genetic risk score” (GRS). For all the population combining European with Chinese samples, the mean of risk score among lung cancer cases (1.04 ± 0.14) was higher than that among cancer-free controls (0.99 ± 0.15), with an average risk score of 1.01 ± 0.15 for all population. We further split the GRS for lung cancer into two subgroups according to its 90% percentage: low risk group (GRS < 1.21), high risk group (GRS ≥ 1.21). Based on the classification of the GRS system, we found that in all the population (9,329 individuals), 8,446 population were classified into the low risk group with 3,792 (44.90%) lung cancer cases and 883 population were classified into the high risk group with 476 (53.91%) lung cancer cases.

**Table 1:** Association of 38SNPs stained by lasso with lung cancer risk in the training data set

SNP	Position	Allele^a^	MAF^b^	*P* (HWE)^b^	OR (95% CI)^c^	*P*^c^	*β*^d^	Author	PMID
rs17728461	chr22:30598552	C/G	0.17	0.70	1.37 (1.24–1.51)	8.50E-10	0.0535	Zhibin Hu	21725308
rs465498*	chr5:1325803	A/G	0.16	0.11	0.75 (0.67–0.84)	6.83E-07	0.0523	Zhibin Hu	21725308
rs753955	chr13:24293859	A/G	0.29	0.76	1.23 (1.13–1.35)	1.33E-06	0.0482	Zhibin Hu	21725308
rs2895680	chr5:146644115	T/C	0.28	0.62	1.21 (1.11–1.32)	1.04E-05	0.0415	Dong J	22797725
rs12296850*	chr12:100820085	A/G	0.25	0.14	0.82 (0.75–0.90)	3.09E-05	0.0402	Dong J	23341777
rs4488809	chr3:189356261	C/T	0.47	1.00	1.21 (1.12–1.31)	2.39E-06	0.0375	Zhibin Hu	21725308
rs2736100	chr5:1286516	A/C	0.41	0.30	1.20 (1.11–1.30)	8.84E-06	0.0374	Chen XF	22370939
rs9439519	chr1:5364634	T/C	0.27	0.93	1.18 (1.08–1.29)	2.18E-04	0.0361	Dong J	22797725
rs383362	chr16:79245820	G/T	0.15	0.62	1.17 (1.05–1.30)	3.97E-03	0.0357	Huang D	22693020
rs6573*	chr1:112255389	C/A	0.13	0.47	0.82 (0.73–0.93)	1.27E-03	0.0346	Zu Y	23232114
rs247008*	chr5:131447104	G/A	0.47	0.06	0.83 (0.77–0.90)	6.27E-06	0.0343	Dong J	22797725
rs4809957	chr20:52771171	G/A	0.35	0.20	1.18 (1.09–1.28)	7.11E-05	0.0341	Dong J	22797725
rs4246215*	chr11:61564299	G/T	0.41	0.85	0.82 (0.76–0.89)	2.04E-06	0.0335	Ming Yang	19618370
rs1663689*	chr10:9025195	T/C	0.42	0.97	0.85 (0.79–0.92)	8.03E-05	0.0313	Dong J	22797725
rs7086803	chr10:114498476	G/A	0.28	0.62	1.16 (1.06–1.26)	1.06E-03	0.0297	Lan Q	23143601
rs4083914	chr6:153427706	G/C	0.14	0.19	1.16 (1.04–1.29)	7.36E-03	0.0293	Li H	23228068
rs2286455*	chr4:16020162	C/T	0.23	0.14	1.15 (1.05–1.26)	3.70E-03	0.0284	Mei Cheng	23715500
rs3764340	chr16:78466437	C/G	0.07	1.00	1.20 (1.04–1.39)	0.012	0.0283	Huang D	22693020
rs36600	chr22:30337586	C/T	0.09	0.82	1.39 (1.22–1.58)	8.38E-07	0.0281	Zhibin Hu	21725308
rs842461	chr3:195535614	T/G	0.27	0.27	1.18 (1.09–1.29)	1.19E-04	0.0253	Zili Zhang	24204934
rs2285053	chr16:55512377	C/T	0.24	0.25	0.90 (0.82–0.99)	0.029	0.0247	GA Patricia	22455335
rs2131877*	chr3:194858374	A/G	0.44	0.07	0.91 (0.84–0.99)	0.025	0.0240	Kyong-Ah Yoon	20876614
rs1801133	chr1:11856378	G/A	0.44	0.36	1.16 (1.07–1.26)	1.76E-04	0.0232	Lian-Hua Cui	21342495
rs3866958*	chr17:19281006	C/A	0.15	0.44	0.87 (0.78–0.97)	0.015	0.0225	Fuman Qiu	23804708
rs1800625	chr6:32152442	A/G	0.13	0.75	1.12 (1.00–1.26)	0.046	0.0218	Wang X	23071492
rs9387478*	chr6:117786180	C/A	0.5	0.86	0.91 (0.84–0.98)	0.013	0.0216	Lan Q	23143601
rs743572	chr10:104597152	G/A	0.4	1.00	1.09 (1.01–1.18)	0.026	0.0209	Zhang Y	22658813
rs4291	chr17:61554194	A/T	0.37	0.08	1.10 (1.02–1.20)	0.015	0.0208	Gao Min	22538550
rs10845498*	chr12:12394574	A/G	0.18	0.11	0.89 (0.80–0.98)	0.023	0.0202	Dehou Deng	24843317
rs7326277*	chr13:28876214	T/C	0.33	0.65	0.91 (0.84–0.99)	0.038	0.0189	Wang H	24891316
rs931127*	chr11:65405300	G/A	0.48	0.08	0.91 (0.84–0.99)	0.028	0.0189	Chenli Xie	23661532
rs2016520	chr6:35378778	T/C	0.27	0.47	1.10 (1.01–1.20)	0.037	0.0161	Eric A. Engels	17596594
rs25406*	chr20:5099636	G/A	0.36	0.56	0.91 (0.84–0.99)	0.025	0.0158	J.A Doherty	23565320
rs2240688*	chr4:15970349	T/G	0.26	0.58	0.91 (0.83–1.00)	0.040	0.0134	Mei Cheng	23715500
rs34843907	chr6:32610059	G/T	0.33	0.39	1.09 (1.00–1.18)	0.041	0.0121	Takashi Kohno	20061363
rs2070600*	chr6:32151443	C/T	0.23	0.29	0.91 (0.83–1.00)	0.046	0.0109	Wang X	23071492
rs189037	chr11:108093833	G/A	0.43	0.07	1.08 (1.00–1.18)	0.049	0.0080	Jing Liu	25541996
rs3817963	chr6:32368087	T/C	0.25	0.07	1.08 (0.99–1.18)	0.078	0.0075	Shiraishi K	22797724

^a^Allele means the change from major allele to minor allele;

^b^Minor allele frequency among controls; HWE among controls;

^c^Logistic regression with adjustment for age, sex, pack year and PCA1;

^d^The coefficient derived from LASSO by adjusting age, sex, smoking statue and PCA1,* means the β coefficient was transformed into the reverse correspond to the risk allele.

### Cumulative effects of genetic and environmental factors with lung cancer

The odds ratios for lung cancer were examined by percentiles of GRS and the total effect combining the smoking statue. In the discovery stage, the estimated OR of subjects in the upper quartile GRS was 4.64 (95% CI: 3.87–5.56) compared to the low quartile (*P* for trend: 7.52E–69). When combined the smoking factor, we found that the risk increases more obviously (*P* for trend: 5.41E–94, Table 2). In addition, this trend was validated in the external data, the risk for lung cancer increased 4.36 times when combined smoking factor with GRS (*P* for trend: 1.81E–53).

**Table 2:** Cumulative effects of associated SNPs and environmental risk factors on the risk of lung cancer

		Case (%)	Control (%)	OR (95% CI)^b^	*P*^b^	*P* for trend
		4268	5061			
**Training set**						
GRS^a^						
	0 (< Q25)	251 (10.77)	775 (25.19)	1		
	1 (Q25–Q50)	430 (18.45)	768 (24.96)	1.80 (1.48–2.19)	2.85E-09	
	2 (Q50–Q75)	590 (25.31)	761 (24.73)	2.48 (2.05–2.99)	3.14E-21	
	3 (≥ Q75)	1060 (45.47)	773 (25.12)	4.64 (3.87–5.56)	4.04E-62	7.52E-69
Smoke + GRS						
	0 (< Q25)	204 (8.75)	773 (25.12)	1		
	1 (Q25–Q50)	337 (14.45)	767 (24.93)	1.78 (1.44–2.20)	1.22E-07	
	2 (Q50–Q75)	557 (23.90)	768 (24.96)	2.99 (2.43–3.66)	1.28E-25	
	3 (≥ Q75)	1233 (52.90)	769 (24.99)	7.01 (5.72–8.58)	3.54E-79	5.41E-94
**Testing set^c^**						
GRS						
	0 (< Q25)	363 (18.74)	496 (25.00)	1		
	1 (Q25–Q50)	442 (22.82)	494 (24.90)	1.19 (0.98–1.46)	7.77E-02	
	2 (Q50–Q75)	531 (27.41)	496 (25.00)	1.50 (1.23–1.82)	4.23E-05	
	3 (≥ Q75)	601 (31.03)	498 (25.10)	1.66 (1.37–2.01)	2.08E-07	1.68E-08
Smoke + GRS						
	0 (< Q25)	148 (7.64)	496 (25.00)	1		
	1 (Q25–Q50)	388 (20.03)	493 (24.85)	2.67 (2.12–3.36)	4.31E-17	
	2 (Q50–Q75)	625 (32.27)	497 (25.05)	4.35 (3.48–5.43)	1.50E-38	
	3 (≥ Q75)	764 (39.44)	493 (24.85)	5.36 (4.30–6.68)	2.36E-50	1.81E-53
**All**						
GRS						
	0 (< Q25)	536 (12.56)	1268 (25.05)	1		
	1 (Q25–Q50)	944 (22.12)	1266 (25.01)	1.70 (1.48–1.94)	2.35E-14	
	2 (Q50–Q75)	1271 (29.78)	1264 (24.98)	2.17 (1.90–2.47)	4.62E-30	
	3 (≥ Q75)	1517 (35.54)	1263 (24.96)	2.31 (2.02–2.64)	1.51E-34	1.31E-34
Smoke + GRS						
	0 (< Q25)	390 (9.14)	1256 (24.82)	1		
	1 (Q25–Q50)	728 (17.06)	1272 (25.13)	1.91 (1.65–2.22)	5.43E-18	
	2 (Q50–Q75)	1223 (28.66)	1260 (24.90)	3.39 (2.93–3.92)	2.31E-61	
	3 (≥ Q75)	1915 (44.87)	1268 (25.05)	5.38 (4.66–6.21)	4.40E-116	4.27E-134

^a^GRS means the genetic risk score with adjustment for age, sex, smoking statue and PCA1;

^b^Adjust for age, sex and PCA1;

^c^For the testing set (the EAGLE study), the smoking status has five missing data.

### Discrimination performance

**Figure 1:**
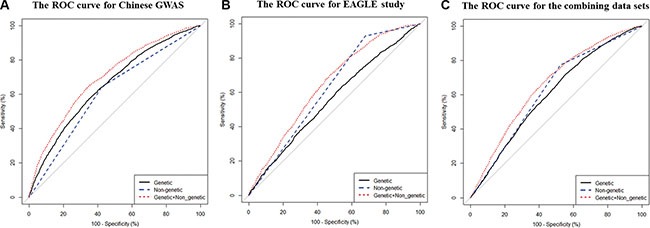
The area under curves (AUCs) for lung cancer risk predicting models calculated by risk score method in the two data sets (A) For Chinese GWAS; (B) For the EAGLE study; (C) For the combined data set

To further assess the discriminative accuracy of the model, we measured the area under curves by C-statistic (Table 3). We found that the model based only on the smoking factor has low discriminatory accuracy in the training data set (AUC = 0.610, Table 3). However, when combining the genetic factors, the performance improves (AUC = 0.697, Table 3, Figure 1A), whether in squamous cell carcinoma, adenocarcinoma or other type of lung cancer (Supplementary Figure S2A–S2C)). Similar performance was also derived among testing samples [*C* statistics: 0.625 (95% CI: 0.613–0.637) *vs.* 0.647 (95% CI: 0.630–0.664), *P* = 0.004, Figure 1B] and combining the two data sets [*C* statistics: 0.625 (95% CI: 0.615–0.634) *vs.*0.658 (95% CI: 0.647–0.669), *P* < 0.001, Figure 1C]. We used the Hosmer-Lemeshow goodness-of-fit test to assess the extended model, indicating that it was an adequate model with *P* value > 0.05 (Table 3). In addition, we found that the genetic model performed moderately with an AUC of 0.604 among non-smokers in the two data sets (Supplementary Figure S2D).

**Table 3:** Area under curves (AUC) as a measure of predictive strength for risk-prediction models based on different indicators

	AUC	95% CI	*P*_AUC_	*P*^a^
**NJMU GWAS**				
Epidemiologic model	0.61	0.597–0.623	1	
Genetic model	0.653	0.639–0.668	< 0.001	
The extended model	0.697	0.683–0.711	< 0.001	0.483
**The EAGLE study**				
Epidemiologic model	0.625	0.613–0.637	1	
Genetic model	0.558	0.540–0.576	< 0.001	
The extended model	0.647	0.630–0.664	0.004	0.662
**The two data sets**				
Epidemiologic model	0.625	0.615–0.634	1	
Genetic model	0.604	0.593–0.616	< 0.001	
The extended model	0.658	0.647–0.669	< 0.001	0.792

^a^Calculated by Hosmer-Lemeshow test.

## DISCUSSION

In this study involving 4,268 lung cancer cases and 5,061 cancer-free controls, 38 of 241 SNPs identified systematically by previous studies were used to calculate genetic risk score. Risk assessment models combining the genetic variants and smoking factor were a good tool to predict the risk value for lung cancer. In our present study, we find that the model with only the smoking factor shows low discriminatory accuracy (AUC = 0.610, in the discovery data set). However, when we plus a genetic risk score based on 38 SNPs into the model, the AUC increases to 0.697 (*P* < 0.001), indicating that genetic predictors could improve the discriminatory ability of the traditional risk model. Furthermore, these results were validated in the external data set EAGLE study and the combined data sets, which mean this risk prediction model can be applied in the European population directly.

Risk prediction models have improved our ability of diagnosis, treatment, and even prevention for diseases by screening high-risk individuals [15]. Recently, a lot of risk prediction models about lung cancer have been developed, such as Bach, LLP and Etzel models [16–18], but most predictors focused on traditional factors (age, smoking status, family history, occupational exposure and so on) with a moderate predictive ability (AUC: 0.55–0.70). As we all know, these models were constructed based on the European population, wondering whether that can be applied in the Chinese population directly. In addition, genetic information might be used to improve the prediction accuracy of above models which offer the stability of the risk prediction during the individual lifetime.

Many studies have indicated that genetic variants might play an important role for lung cancer risk [19, 20]. So far, GWAS have identified some important lung cancer susceptibility loci: 22q12 (*MTMR3-HORMAD2*), 3q28 (*TP63*) and 5p15 (*TERT-CLPM1L*) [21–23]. Of the 38 SNPs evaluating the clinical utility in the present study, we found that the top 3 genetic variants with a strong signal depending on the β coefficient were mainly located on these loci. The variant rs17728461 included in our model was located in the intron at 22q12.2, a region which includes the HORMA domain-containing protein 2 (*HORMAD2*). The putative functions of the gene include mitotic checkpoints, chromosome synapsis and DNA repair. And also *HORMAD2* has been identified as a CT (cancer-testis) gene by silico methods [21] which indicate that *HORMAD2* may contribute to the lung adenocarcinoma risk [24]. The SNP rs753955 was located in the intron at 13q12.12 region between *MIPEP* and *TNFRSF19* identified as a risk locus of lung cancer by recent GWA studies [21]. The protein of MIPEP is primarily involved in the maturation of oxidative phosphorylation –related proteins and TNFRSF19 which is a member of the TNF-receptor superfamily actives JNK signaling pathway when overexpressed in cells.

The 5p15 region containing *TERT* and *CLPM1L* genes was thought to be related to lung cancer risk by recent GWA studies in European [22, 25–27], East Asian and African – American populations [23, 28]. The marker of lung cancer rs465498 [21] located in *CLPM1L* encoding the cleft lip and palate-associated transmembrane 1 like protein had strong contribution to our genetic risk model. Of the 38 SNPs included in our model, the β coefficient calculated by LASSO was from 0.0075 to 0.0535, this suggested that the genetic variants only show a small contribution risk in our risk prediction model when considered alone, and are of little value in the application.

Recently, several studies have been published that a better prediction could be achieved if we combined genetic determinants into traditional approaches to assess an individual risk [10–12]. Weissfeld *et al.* [12] constructed a lung cancer risk prediction model and found that the area under the receiver operator characteristic curve improved from 0.717 to 0.725 when adding GWAS susceptibility regions to an age and smoking risk factor-only model. However, only six SNPs were included into risk prediction model. In our current study, more genetic variants were incorporated even though the performance of the risk assessment model was limited. The AUC increased from 0.610 to 0.697 when adding the 38-GRS to the smoking risk factor-only model in our discovery set.

This study has several notable strengths. First, this risk prediction model developed in our Chinese population and externally validated in both European and Asian populations, which means this model has a good extrapolation. Therefore, we are able to use the model to predict the risk of lung cancer among different ethnic populations. Furthermore, to our knowledge, this study constructed the risk prediction model by the system of screening and evaluating genetic susceptibility from the past papers that has high predictive ability accuracy. The 38-SNP GRS has public health utility by screening high-risk individuals. As shown in Table 2, the risk for lung cancer in the highest GRS increases 131% compared with the lowest for combing Chinese and European populations. It can help us make a better decision about whether to be screened by locating themselves along the spectrum of lung cancer risk [29]. In addition, for never smokers the predictive value of the genetic model was moderate and for all population our risk model combining genetic variants with smoking factor can improve the ability of prediction significantly. Therefore, we use the risk model with GRS combining multiple loci to improve the identification of persons at high risk for lung cancer.

However, some limitations in our study should be noted. This research only included smoking statue as the traditional non-genetic factors, which led to the poor discrimination. Some other studies, such as Spitz MR et al. were concentrated on the data of other clinical information such as family history of lung cancer and asbestos-exposure besides of tobacco smoke [18]. Moreover, GWAS and candidate-gene studies mainly focus on common proxy SNPs with many rare and low frequency loci or copy number variants for lung cancer to be discovered. Combing these additional variants might result in improvement in classification of lung cancer risk.

In conclusion, this is the first attempt to explore the risk predictive effects of genetic risk factors associated with lung cancer in both Chinese and European populations. In our study, 38 genetic variants identified by GWAS or candidate-gene strategies were used to construct the risk prediction models. Risk predictive models that incorporate both a genetic risk score based on these SNPs and smoking factors for lung cancer may be useful in identifying high-risk populations for targeted cancer prevention. More genetic risk variants and other epidemiological factors should be well evaluated and incorporated into the risk-predicting models to improve the ability of personalized risk assessment.

## MATERIALS AND METHODS

### Study subjects

For the training set, derived from a lung cancer GWAS in NJMU (Nanjing Medical University) [21, 30] 2,331 lung cancer cases and 3,077 cancer-free controls were enrolled in this model; for the testing set, 1,937 cases and 1,984 controls were used to validate the risk prediction model, which were derived from NCI GWAS: Environment and Genetics in Lung Cancer Etiology (EAGLE) [25].

Subjects used in the two stages were genotyped using the Affymetrix Genome-Wide Human SNP Array 6.0 microarray [21, 30] and Illumina Human660W-Quad v1.0 DNA Analysis BeadChip platform (Illumina, San Diego, CA, USA) [25] respectively. To facilitate further analysis, imputation analysis were performed by IMPUTE2 software taking 1000 Genomes Project data (Phase III) as reference set. We implemented a 4-Mb sliding window to impute across the genome, resulting in 744 windows. A pre-phasing strategy with SHAPEIT software version 2 was adopted to improve the imputation performance. The phased haplotypes from SHAPEIT were fed directly into IMPUTE2.

### Literature review strategy and SNP selection

Eligible studies were identified by performing a literature search on the PubMed (last search in June 30, 2015 by using the following keywords: “Lung cancer AND polymorphism”. Furthermore, we scrutinized the full text of each paper to follow these criteria (Supplementary Figure S1): i) The studies were about human population and the publishing language was English; ii) these papers had an observational (case-control or cohort) study design (the sample size was at least 500 vs 500); iii) the authors offered odds ratios (ORs) and their 95% confidence intervals (CIs) of the relevant SNPs. In cases where the studies met the inclusive criteria, 241 genetic variants in 124 papers were selected in our study.

We screened all the SNPs based on the relevant papers mentioned above followed three criteria (Supplementary Figure S1): (i) SNP with imputed INFO ≥ 0.8; (ii) minor allele frequency (MAF) in controls ≥ 0.05 and *P* value for HWE in controls ≥ 0.05; (iii) only the SNP with the lowest *P* value was selected when multiple SNPs were observed in a moderate or strong linkage disequilibrium (LD) (r^2^ ≥ 0.5, LD window: 200 kb). In total, 148 SNPs passed quality control.

### Public database

PLINK 1.07, http://pngu.mgh.harvard.edu/˜purcell/plink/index.shtml;

R software 3.1.1, http://www.cran.r-project.org/;

IMPUTE2 software, http://mathgen.stats.ox.ac.uk/impute/impute_v2.html;

1000 Genomes Project, http://www.1000genomes.org/;

SHAPEIT software version 2,

http://mathgen.stats.ox.ac.uk/genetics_software/shapeit/shapeit.html;

Environment and Genetics in Lung Cancer Etiology (EAGLE),

http://www.ncbi.nlm.nih.gov/projects/gap/cgi-bin/study.cgi?study_id=phs000093.v2.p2.

### Statistical analyses

We used the NJMU GWAS samples as the training set to guide model development and the European samples as the validation set to assess the accuracy of the risk model. Four steps were performed to develop the risk model (listed in Supplementary Figure S1). Step I SNPs screening. 40 significant SNPs (*P* < 0.05) were picked out using PLINK 1.07 through univariate analysis. Further, we used the Least Absolute Shrinkage and Selection Operator (LASSO) penalized regression model in the discovery stage (2,331 cases/3,077 controls) and 38 genetic variants were included in our predictive models. Step II Model construction. To evaluate the contribution of the genetic factors, we conducted 2 risk models, one was the epidemiologic model (containing smoking factor only) and the other was the extended model (adding genetic variants evaluated by genetic risk score). In this model, “genetic risk score” (GRS) means the cumulative effect of multiple genetic risk variants as follows:

$$\overset{k}{\underset{i=1}{\sum }}\beta i\text{*SNPi}$$

Where k is the number of SNPs replicates in the study; SNP_i_ is the number of the risk alleles (0, 1, 2); β_*i*_ is the regression coefficient for SNP_i_, which was derived by using LASSO selection. It's worth noting that we rescaled the weighted score to reflect the number of risk allele: each point of the genetic risk score corresponded to one risk allele. Step III Model evaluation. Model discrimination was evaluated by receiver-operator characteristic curves (ROC) and the *C* statistics. A nonparametric approach was used to compare the area under the receiver operating characteristic (ROC) curves (AUC) for the two models [31]. To quantify discriminatory improvement for models with and without the genetic risk score, we also set a cut-off value of the genetic risk score (GRS). Step IV Model validation. We validated the risk model in the EAGLE samples (1,937 cases vs 1,984 controls) with the same risk predictors and evaluation strategies.

All statistical analyses were performed with PLINK 1.07 and R software (version 3.1.1; The R Foundation for Statistical Computing). *P* < 0.05 was used as the criterion of statistical significance and all statistical tests were two sided.

## SUPPLEMENTARY FIGURES AND TABLE



## References

[1] Ferlay J, Soerjomataram I, Dikshit R, Eser S, Mathers C, Rebelo M, Parkin DM, Forman D, Bray F (2015). Cancer incidence and mortality worldwide: sources, methods and major patterns in GLOBOCAN 2012. Int J Cancer.

[2] Peto R, Darby S, Deo H, Silcocks P, Whitley E, Doll R (2000). Smoking, smoking cessation, and lung cancer in the UK since 1950: combination of national statistics with two case-control studies. Bmj.

[3] Paynter NP, Chasman DI, Pare G, Buring JE, Cook NR, Miletich JP, Ridker PM (2010). Association between a literature-based genetic risk score and cardiovascular events in women. Jama.

[4] Zheng W, Wen W, Gao YT, Shyr Y, Zheng Y, Long J, Li G, Li C, Gu K, Cai Q, Shu XO, Lu W (2010). Genetic and clinical predictors for breast cancer risk assessment and stratification among Chinese women. J Natl Cancer Inst.

[5] Wacholder S, Hartge P, Prentice R, Garcia-Closas M, Feigelson HS, Diver WR, Thun MJ, Cox DG, Hankinson SE, Kraft P, Rosner B, Berg CD, Brinton LA (2010). Performance of common genetic variants in breast-cancer risk models. N Engl J Med.

[6] Johansson M, Holmstrom B, Hinchliffe SR, Bergh A, Stenman UH, Hallmans G, Wiklund F, Stattin P (2012). Combining 33 genetic variants with prostate-specific antigen for prediction of prostate cancer: longitudinal study. Int J Cancer.

[7] Hsu FC, Sun J, Zhu Y, Kim ST, Jin T, Zhang Z, Wiklund F, Kader AK, Zheng SL, Isaacs W, Gronberg H, Xu J (2010). Comparison of two methods for estimating absolute risk of prostate cancer based on single nucleotide polymorphisms and family history. Cancer Epidemiol Biomarkers Prev.

[8] Hsu FC, Lindstrom S, Sun J, Wiklund F, Chen SH, Adami HO, Turner AR, Liu W, Balter K, Kim JW, Stattin P, Chang BL, Isaacs WB (2008). A multigenic approach to evaluating prostate cancer risk in a systematic replication study. Cancer Genet Cytogenet.

[9] Talmud PJ, Hingorani AD, Cooper JA, Marmot MG, Brunner EJ, Kumari M, Kivimaki M, Humphries SE (2010). Utility of genetic and non-genetic risk factors in prediction of type 2 diabetes: Whitehall II prospective cohort study. Bmj.

[10] Li H, Yang L, Zhao X, Wang J, Qian J, Chen H, Fan W, Liu H, Jin L, Wang W, Lu D (2012). Prediction of lung cancer risk in a Chinese population using a multifactorial genetic model. BMC Med Genets.

[11] Spitz MR, Amos CI, Land S, Wu X, Dong Q, Wenzlaff AS, Schwartz AG (2013). Role of selected genetic variants in lung cancer risk in African Americans. J Thorac Oncol.

[12] Weissfeld JL, Lin Y, Lin HM, Kurland BF, Wilson DO, Fuhrman CR, Pennathur A, Romkes M, Nukui T, Yuan JM, Siegfried JM, Diergaarde B (2015). Lung Cancer Risk Prediction Using Common SNPs Located in GWAS-Identified Susceptibility Regions. J Thorac Oncol.

[13] Gulati S, Mulshine JL (2014). Lung cancer screening guidelines: common ground and differences. Transl Lung Cancer Res.

[14] Zhao SJ, Wu N (2015). Early detection of lung cancer: Low-dose computed tomography screening in China. Thorac Cancer.

[15] Collins FS, McKusick VA (2001). Implications of the Human Genome Project for medical science. Jama.

[16] Bach PB, Kattan MW, Thornquist MD, Kris MG, Tate RC, Barnett MJ, Hsieh LJ, Begg CB (2003). Variations in lung cancer risk among smokers. J Natl Cancer Inst.

[17] Cassidy A, Myles JP, van Tongeren M, Page RD, Liloglou T, Duffy SW, Field JK (2008). The LLP risk model: an individual risk prediction model for lung cancer. Br J Cancer.

[18] Spitz MR, Hong WK, Amos CI, Wu X, Schabath MB, Dong Q, Shete S, Etzel CJ (2007). A risk model for prediction of lung cancer. J Natl Cancer Inst.

[19] Matakidou A, Eisen T, Houlston RS (2005). Systematic review of the relationship between family history and lung cancer risk. Br J Cancer.

[20] Zhang Y, Shu XO, Gao YT, Ji BT, Yang G, Li HL, Kilfoy B, Rothman N, Zheng W, Chow WH (2007). Family history of cancer and risk of lung cancer among nonsmoking Chinese women. Cancer Epidemiol Biomarkers Prev.

[21] Hu Z, Wu C, Shi Y, Guo H, Zhao X, Yin Z, Yang L, Dai J, Hu L, Tan W, Li Z, Deng Q, Wang J (2011). A genome-wide association study identifies two new lung cancer susceptibility loci at 13q12.12, 22q12.2 in Han Chinese. Nat Genet.

[22] McKay JD, Hung RJ, Gaborieau V, Boffetta P, Chabrier A, Byrnes G, Zaridze D, Mukeria A, Szeszenia-Dabrowska N, Lissowska J, Rudnai P, Fabianova E, Mates D (2008). Lung cancer susceptibility locus at 5p15.33. Nat Genet.

[23] Lan Q, Hsiung CA, Matsuo K, Hong YC, Seow A, Wang Z, Hosgood HD, Chen K, Wang JC, Chatterjee N, Hu W, Wong MP, Zheng W (2012). Genome-wide association analysis identifies new lung cancer susceptibility loci in never-smoking women in Asia. Nat Genet.

[24] Liu M, Chen J, Hu L, Shi X, Zhou Z, Hu Z, Sha J (2012). HORMAD2/CT46. 2, a novel cancer/testis gene, is ectopically expressed in lung cancer tissues. Mol Hum Reprod.

[25] Landi MT, Chatterjee N, Yu K, Goldin LR, Goldstein AM, Rotunno M, Mirabello L, Jacobs K, Wheeler W, Yeager M, Bergen AW, Li Q, Consonni D (2009). A genome-wide association study of lung cancer identifies a region of chromosome 5p15 associated with risk for adenocarcinoma. Am J Hum Genet.

[26] Wang Y, Broderick P, Webb E, Wu X, Vijayakrishnan J, Matakidou A, Qureshi M, Dong Q, Gu X, Chen WV, Spitz MR, Eisen T, Amos CI (2008). Common 5p15.33 and 6p21.33 variants influence lung cancer risk. Nat Genet.

[27] Rafnar T, Sulem P, Stacey SN, Geller F, Gudmundsson J, Sigurdsson A, Jakobsdottir M, Helgadottir H, Thorlacius S, Aben KK, Blondal T, Thorgeirsson TE, Thorleifsson G (2009). Sequence variants at the TERT-CLPTM1L locus associate with many cancer types. Nat Genet.

[28] Hsiung CA, Lan Q, Hong YC, Chen CJ, Hosgood HD, Chang IS, Chatterjee N, Brennan P, Wu C, Zheng W, Chang GC, Wu T, Park JY (2010). The 5p15. 33 locus is associated with risk of lung adenocarcinoma in never-smoking females in Asia. PLoS Genet.

[29] Bach PB, Schrag D (2002). Risk charts: putting cancer in context. J Natl Cancer Inst.

[30] Dong J, Hu Z, Wu C, Guo H, Zhou B, Lv J, Lu D, Chen K, Shi Y, Chu M, Wang C, Zhang R, Dai J (2012). Association analyses identify multiple new lung cancer susceptibility loci and their interactions with smoking in the Chinese population. Nat Genet.

[31] DeLong ER, DeLong DM, Clarke-Pearson DL (1988). Comparing the areas under two or more correlated receiver operating characteristic curves: a nonparametric approach. Biometrics.

