# Supplementary material for: Risk assessment models for genetic risk predictors of lung cancer using two-stage replication for Asian and European populations
Source: Oncotarget. 2016 Jul 5;8(33):53959–67. doi: 10.18632/oncotarget.10403 (PMC5589554; doi:10.18632/oncotarget.10403)
Supplement: Supplementary file 1 [file oncotarget-08-53959-s001.pdf]

Risk assessment models for genetic risk predictors of lung cancer using two-stage replication for Asian and European populations

Supplementary Materials

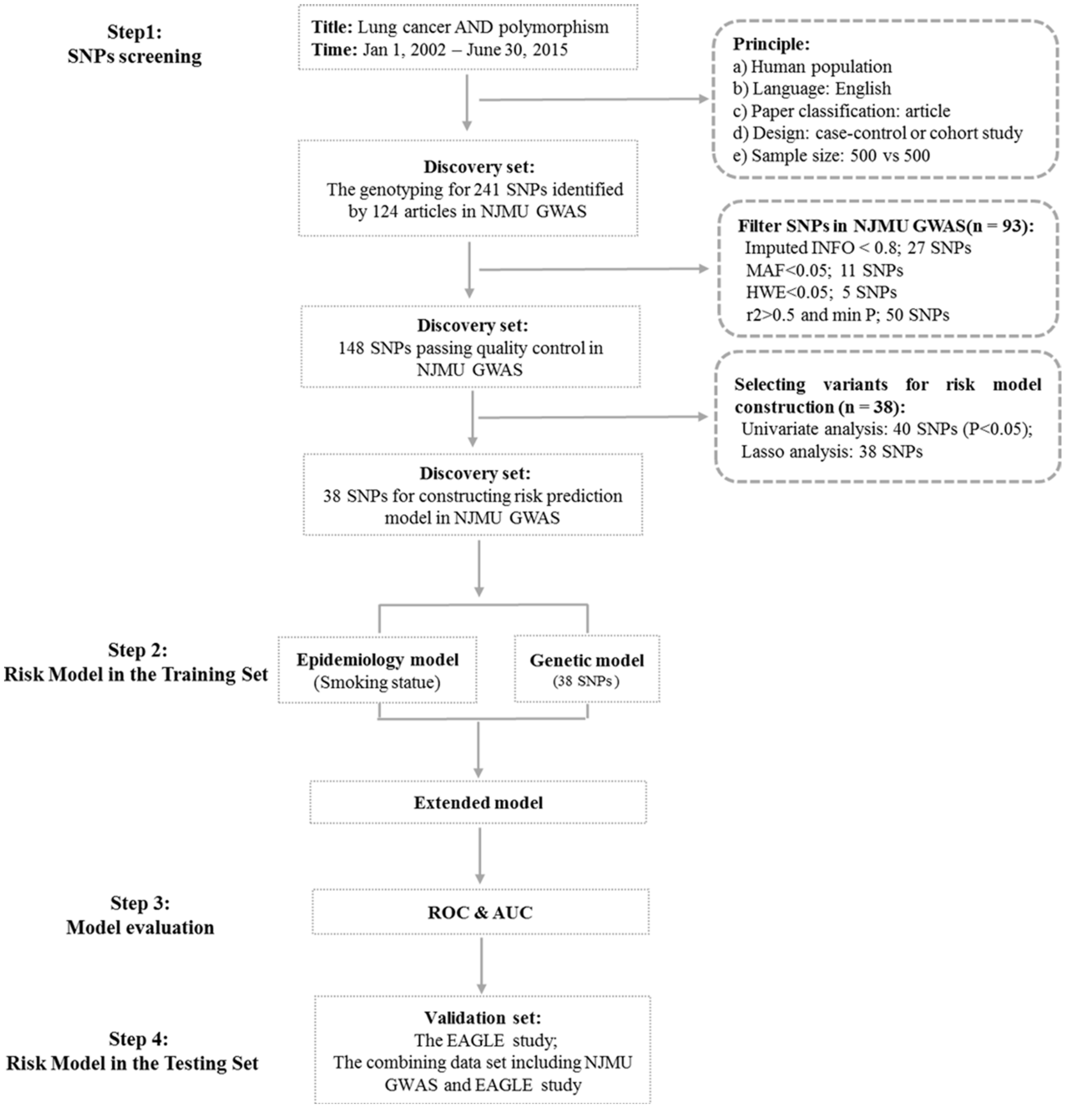

Supplementary Figure S1: The flow chart for genetic variants screening and risk prediction models construction.

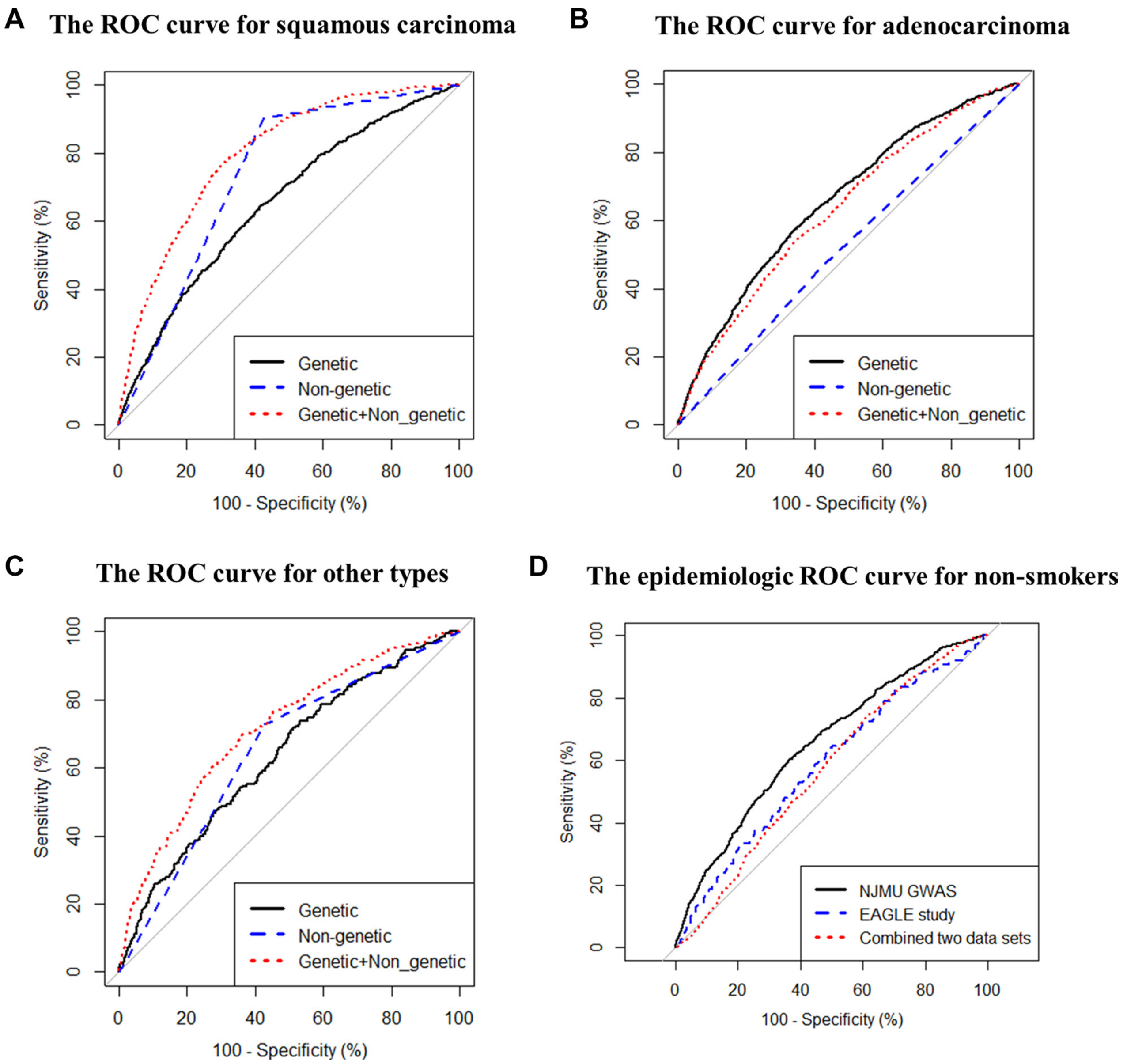

Supplementary Figure S2: The area under curves (AUCs) for the different histology of lung cancer risk predicting models calculated by risk score method in discovery data set and the genetic model among the non-smokers in the two data sets. (**A**) For lung squamous carcinoma; (**B**) For lung adenocarcinoma; (**C**) For other histology of lung cancer; (**D**) For genetic model among the non-smokers in the two data sets.

Supplementary Table S1: Distribution of demographic characteristics for cases and controls included in the study

	NJMU GWAS		EAGLE study[a]		The two data sets	
	Case 2331 (%)	Control 3077 (%)	Case 1937 (%)	Control 1984 (%)	Case 4268 (%)	Control 5061 (%)
**Age**						
< 60	1111 (47.66)	1429 (46.44)	423 (21.84)	502 (25.30)	1534 (35.94)	1931 (38.16)
60–70	799 (34.28)	1045 (33.96)	772 (39.86)	806 (40.63)	1571 (38.81)	1851 (36.57)
≥ 70	421 (18.06)	603 (19.60)	742 (38.30)	676 (34.07)	1163 (27.25)	1279 (25.27)
**Sex**						
Male	1711 (73.40)	2086 (67.79)	1532 (79.09)	1519 (76.56)	3243 (75.98)	3605 (71.23)
Female	620 (26.60)	991 (32.21)	405 (20.91)	465 (23.44)	1025 (24.02)	1456 (28.77)
**Smoke status**						
Never	838 (35.95)	1755 (57.04)	138 (7.12)	636 (32.06)	976 (22.87)	2391 (47.24)
Ever	1493 (64.05)	1322 (42.96)	1787 (92.26)	1343 (67.62)	3280 (76.85)	2665 (52.66)

[a]The smoking status has 12 and 5 missing data among the EAGLE study respectively.
